# An Adaptive Clear High-Dynamic Range Fusion Algorithm Based on Field-Programmable Gate Array for Real-Time Video Stream

**DOI:** 10.3390/s26020577

**Published:** 2026-01-15

**Authors:** Hongchuan Huang, Yang Xu, Tingyu Zhao

**Affiliations:** Zhejiang Key Laboratory of Quantum State Control and Optical Field Manipulation, Department of Physics, Zhejiang Sci-Tech University, Hangzhou 310018, China; 202230106294@mails.zstu.edu.cn (H.H.); 202130105236@mails.zstu.edu.cn (Y.X.)

**Keywords:** image fusion, video stream, clear HDR, bit depth, field programmable gate array

## Abstract

Conventional High Dynamic Range (HDR) image fusion algorithms generally require two or more original images with different exposure times for synthesis, making them unsuitable for real-time processing scenarios such as video streams. Additionally, the synthesized HDR images have the same bit depth as the original images, which may lead to banding artifacts and limits their applicability in professional fields requiring high fidelity. This paper utilizes a Field Programmable Gate Array (FPGA) to support an image sensor operating in Clear HDR mode, which simultaneously outputs High Conversion Gain (HCG) and Low Conversion Gain (LCG) images. These two images share the same exposure duration and are captured at the same moment, making them well-suited for real-time HDR fusion. This approach provides a feasible solution for real-time processing of video streams. An adaptive adjustment algorithm is employed to address the requirement for high fidelity. First, the initial HCG and LCG images are fused under the initial fusion parameters to generate a preliminary HDR image. Subsequently, the gain of the high-gain images in the video stream is adaptively adjusted according to the brightness of the fused HDR image, enabling stable brightness under dynamic illumination conditions. Finally, by evaluating the read noise of the HCG and LCG images, the fusion parameters are adaptively optimized to synthesize an HDR image with higher bit depth. Experimental results demonstrate that the proposed method achieves a processing rate of 46 frames per second for 2688 × 1520 resolution video streams, enabling real-time processing. The bit depth of the image is enhanced from 12 bits to 16 bits, preserving more scene information and effectively addressing banding artifacts in HDR images. This improvement provides greater flexibility for subsequent image processing tasks. Consequently, the adaptive algorithm is particularly suitable for dynamically changing scenarios such as real-time surveillance and professional applications including industrial inspection.

## 1. Introduction

Both highlight and shadow regions perceptible to the human eye cannot be simultaneously captured by cameras through sole adjustment of exposure duration and ISO sensitivity. This limitation is fundamentally attributed to the exceptionally broad luminance range spanning from complete darkness to maximum brightness in natural environments, wherein the preservation of both highlight and shadow details is inevitably compromised by data acquisition systems with limited bit depth [1,2,3,4,5]. Researchers have conducted explorations in different fields from the perspectives of both hardware design and software technology [6]. While it is true that designing sensors capable of capturing higher bit-depth images can solve the problem at its root, such image sensors also imply significantly higher hardware costs. On the other hand, the HDR (High Dynamic Range) fusion algorithm enables the integration of image information with a wide dynamic range into a single image through systematic integration of pre-captured images containing highlight details and shadow details.

Conventional HDR fusion algorithms are generally divided into two stages: the initial acquisition of multiple images with varying exposure durations, followed by computational processing of these captured frames. The majority of conventional HDR fusion methodologies primarily focus on the second stage. For instance, Mertens et al. [7] computed perceptual quality metrics (e.g., saturation, contrast) at each pixel across multi-exposure sequences and employed these metrics to drive exposure blending through weighted fusion. Liu et al. [8] employed dense Scale-Invariant Feature Transform (SIFT) descriptors as activity-level metrics to extract localized structural details from multi-exposure source images. Li et al. [9] decomposed input images into base layers (encoding large-scale luminance variations) and detail layers (capturing high-frequency textures) via two-scale decomposition, followed by a guided filtering-based weighted fusion framework to preserve spatial coherence during layer integration. However, these conventional methods remain constrained by their reliance on pre-captured images with varying exposure durations and timings, rendering them incapable of real-time video stream processing. Furthermore, inevitable object motion during the imaging process results in imperfect spatial registration of pre-acquired images, leading to ghosting artifacts in the fused composite image. Conventional ghosting artifacts suppression algorithms address this issue at the expense of computational efficiency yet merely achieve partial suppression rather than complete elimination. In terms of achieving similar HDR effects, Z. Li et al. [10] proposed a method for automatic exposure correction based on a single input image. However, when parts of the input image are completely black or white, the corrected image cannot fully recover these areas due to the lack of suitable input data for reference. This limitation is inherent to approaches that rely on a single input image. In addition, to achieve favorable HDR fusion results, deep learning approaches have been emerging continuously. Among them, Lucas Nedel Kirsten et al. [11] propose MobileMEF, a novel method for multi-exposure fusion based on an encoder–decoder deep learning architecture with efficient building blocks tailored for mobile devices. Although this method can process 4K-resolution images in less than 2 s, deep learning approaches still remain overly complex and challenging to deploy when real-time processing of more than 40 frames of images to be fused is required.

Clear HDR revolutionizes image acquisition by simultaneously capturing dual-exposure frames—defined by a High Conversion Gain (HCG) and a Low Conversion Gain (LCG)—that share an identical exposure duration and are taken at the same precise moment [12]. This approach completely solves the inherent problems of ghosting artifacts and real-time processing constraints. However, a key limitation of their approach was its inability to account for read noise amplification during processing, thus failing to resolve the persistent banding artifacts in the final image. Furthermore, they neglected environmental illumination variations caused by scene dynamics in video stream acquisition, thereby failing to eliminate overbrightness or underexposure phenomena in composite outputs. Simultaneously, similar to conventional HDR algorithms, this method remains constrained by the requirement to maintain consistent bit depth before and after fusion, which significantly limits its applicability in professional fields that necessitate the preservation of original image data for post-processing workflows.

An optimized algorithm, named the Buffer Optimization Algorithm, is proposed to leverage Clear HDR capability for synthesizing dual co-exposure images (HCG and LCG outputs) through adaptive parameter optimization. A feedback mechanism based on post-fusion metrics—such as mean luminance and read noise distribution—is designed to dynamically adjust fusion weights. This closed-loop control framework enables real-time HDR video stream processing with enhanced dynamic range preservation and computational efficiency, while ensuring robust performance across varying lighting conditions. The design of this adaptive mechanism also constitutes one of the core objectives of the present study.

## 2. Materials and Methods

### 2.1. Clear HDR Fusion Algorithm with Buffer Module

**Figure 1:**
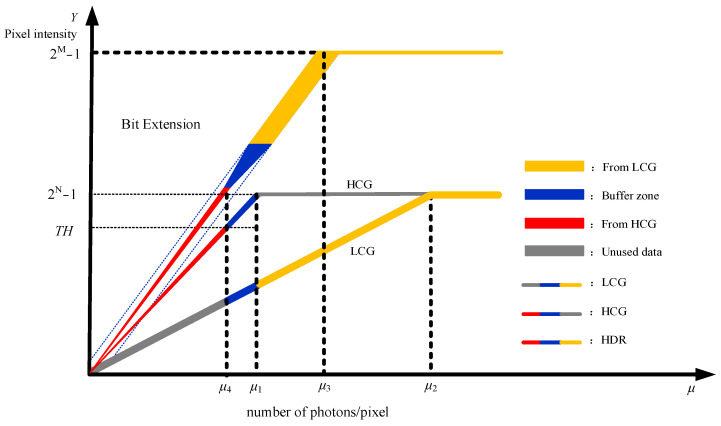
LCG, HCG, and HDR curves.

This subsection describes the Clear HDR fusion model with a buffer module and explains how the threshold-based strategy balances dynamic range extension and noise suppression. Figure 1 illustrates the relationship between the sensor pixel grayscale value and the average number of photons. The solid red and yellow lines in the figure represent the HCG and LCG curves, respectively. It is noted that their maximum values are both 2*^N^* − 1, indicating a bit depth of *N* bits for both. Notice that saturation occurs in the HCG and LCG channels when their respective photon counts exceed *μ*_1_ and *μ*_2_. This overexposure causes the pixel value to clip at its maximum, breaking the linear response and leading to information loss. For photon counts within the interval [*μ*_1_, *μ*_2_], the HCG is saturated while the LCG remains unsaturated. Thus, the unsaturated LCG data can be utilized as compensation data to mitigate the information loss caused by HCG saturation.

Below, we express the HCG and LCG curves shown in Figure 1 with equations. Let analog gains *K*_HCG_ and *K*_LCG_ denote the pixel sensitivity coefficients under HCG and LCG modes, respectively, and *μ* represents the average number of photons per pixel. The expression describing the HCG and LCG curves in Figure 1 is as follows [12]:

(1) $$Y_{\mathrm{HCG}}=\left\{\begin{array}{ll} K_{\mathrm{HCG}}\mu , & \left(K_{\mathrm{HCG}}\mu <2^{N}-1\right)\\ 2^{N}-1 & \left(K_{\mathrm{HCG}}\mu \geq 2^{N}-1\right) \end{array}\right.$$

(2) $$Y_{\mathrm{LCG}}=\left\{\begin{array}{ll} K_{\mathrm{LCG}}\mu , & \left(K_{\mathrm{LCG}}\mu <2^{N}-1\right)\\ 2^{N}-1, & \left(K_{\mathrm{LCG}}\mu \geq 2^{N}-1\right) \end{array}\right.$$

The expression above merely describes the numerical relationship between pixel values and HCG/LCG, although it does not account for the effects of noise. Thus, line thickness is used to represent noise levels in Figure 1, with thicker curves indicating higher noise. Generally, image noise includes shot noise, readout noise, and dark current noise. Among these, dark current noise is negligible in normal imaging scenarios [13] and is thus ignored from this model. Given that readout noise is inversely proportional to gain and remains constant at a fixed gain, while shot noise depends on the pixel value [13], the primary noise source in HCG images is shot noise, whereas readout noise dominates in LCG images. When evaluating the dominant noise source in the fused image, it is primarily attributed to the Bit Extension region highlighted in yellow. This is because shot noise, which is proportional to the standard deviation of pixel values, becomes significantly amplified when the LCG component is scaled to the same gain level as the HCG component. The amplified shot noise in the LCG portion exceeds that in the HCG portion. Conversely, for LCG images, readout noise dominates over shot noise. Thus, the Bit Extension region (yellow) is also dominated by readout noise. Consequently, the primary noise source in the fused image is the amplified read noise from the LCG component. If an HDR image is synthesized by directly superimposing the raw HCG data with the amplified LCG data, the resultant noise profile is inevitably suboptimal. The noise remains relatively low in the HCG segment, as evidenced by the thin red line, but becomes significantly pronounced in the amplified LCG segment, indicated by the thick yellow line. This disparity unavoidably leads to banding artifacts. Therefore, to balance noise amplification and transition smoothness, an adaptive threshold *TH* and a buffer zone are introduced.

As illustrated in Figure 1, HDR data below *TH* adopts the original HCG data, while data above *TH* employs the scaled LCG data, where the scaling factor is the gain ratio between HCG and LCG. The buffer zone is constructed between *TH* and 2*^N^* − 1. By adaptively adjusting *TH*, the proportion of HCG and LCG data within the buffer zone is dynamically optimized, thereby facilitating a smoother transition of the Clear HDR fusion curve from the HCG segment to the Bit Extension segment. The incorporation of the buffer zone not only ensures a more gradual transition in the fusion curve but also reduces the proportion of LCG data involved in fusion, effectively minimizing readout noise.

Let *K*_D_ denotes the digital gain. Based on the above analysis, the fused HDR curve can be expressed as:

(3) $$Y_{\mathrm{HDR}}=\left\{\begin{array}{ll} K_{D}Y_{\mathrm{HCG}}, & \left(K_{D}Y_{\mathrm{HCG}}\leq TH\right)\\ K_{D}\frac{Y_{\mathrm{HCG}}\left(2^{N}-1-Y_{\mathrm{HCG}}\right)+\frac{K_{\mathrm{HCG}}}{K_{\mathrm{LCG}}}Y_{\mathrm{LCG}}\left(Y_{\mathrm{HCG}}-TH\right)}{2^{N}-1-TH}, & \left(TH<K_{D}Y_{\mathrm{HCG}}<2^{N}-1\right)\\ K_{D}\frac{K_{\mathrm{HCG}}}{K_{\mathrm{LCG}}}Y_{\mathrm{LCG}}, & \left(2^{N}-1\leq K_{D}Y_{\mathrm{HCG}}\leq 2^{M}-1\right)\\ 2^{M}-1, & \left(K_{D}Y_{\mathrm{HCG}}\geq 2^{M}-1\right) \end{array}\right.$$

It should be noted that unlike analog gain, digital gain *K*_D_ does not enhance the sensor’s sensitivity; instead, it is a parameter that amplifies pixel values within the algorithm. However, while amplifying pixel values, digital gain also amplifies noise. To ensure the fused HDR image reaches saturation, and *M* represents the bit depth after HDR fusion, which should be no less than the bit depths of LCG and HCG images. Furthermore, since increasing the digital gain does not inherently extend the bit depth, the term *K*_D_*K*_HCG_/*K*_LCG_ in Equation (3) must satisfy Equation (4) when *K*_D_ = 1:

(4) $$K_{D}\frac{K_{\mathrm{HCG}}}{K_{\mathrm{LCG}}}\geq 2^{M-N}$$

Failure to meet this condition will prevent the pixel values of the fused image from attaining their maximum, leading to underexposure.

Since the analog gain *K*_HDR_ is constrained by sensor limitations, it cannot be adjusted indefinitely. When *K*_HDR_ reaches its minimum value but the image remains overexposed, the exposure duration must be reduced to control the brightness of the fused image. Conversely, when *K*_HDR_ attains maximum value yet the fused image is still underexposed, i.e., *K*_HCG_/*K*_LCG_ ≤ 2*^M^*^−*N*^, the deficit in gain must be compensated by the digital gain *K*_D_. In this case, *K*_D_ exceeds 1, globally amplifying the fused image by a factor of *K*_D_ to achieve the desired brightness. When *K*_HDR_ falls between the adjustable maximum and minimum values, the target brightness can be achieved solely by modifying the analog gain *K*_HDR_, with the digital gain *K*_D_ remaining at 1. Thus, the digital gain *K*_D_ can be regarded as a complementary gain.

The constant variation in factors like scene brightness and noise makes it impossible to define a fixed parameter set that is universally optimal under all conditions. Consequently, an adaptive adjustment algorithm for gain and threshold values is required to cope with variations in scene brightness and noise characteristics. Based on this observation, the adaptive adjustment strategies for Clear HDR parameters are introduced in the next subsection.

### 2.2. Adaptive Adjustment of Clear HDR Parameters

In the proposed framework, the Clear HDR fusion parameters mainly include gain-related parameters and the fusion threshold. Accordingly, adaptive gain adjustment and adaptive threshold selection are discussed separately in the following subsections.

#### 2.2.1. Adaptive Gain

As derived from Equation (3), the overall brightness of the fused image is determined by the analog gains *K*_HCG_ and *K*_LCG_, along with the digital gain *K*_D_. Since *K*_LCG_ influences *Y*_LCG_, it is generally held constant, leaving *K*_HCG_ and *K*_D_ as the primary adjustable parameters. Analog gain is prioritized in adaptive adjustment due to its characteristic of not amplifying overall noise.

Let *B*_HCG_ and *K*_HCG_ denote the current brightness and gain in HCG mode, respectively. Let *B*_HDR_ and *K*_HDR_ denote the expected brightness of the fused HDR image and the overall gain required to achieve that brightness (including both digital gain and analog gain), respectively. *P* denotes the number of pixels in the target region. The brightness *B* can be obtained by calculating the average grayscale value of the target region

(5) $$B=\frac{\sum_{k=1}^{P}Y}{P}$$

During the initial HDR fusion, since the brightness of the fused image is uncertain, the digital gain *K*_D_ can be temporarily set to 1. The target gain *K*_HDR_ can be derived using the following relationship

(6) $$\frac{B_{\mathrm{HDR}}}{K_{\mathrm{HDR}}}=\frac{B_{\mathrm{HCG}}}{K_{D}K_{\mathrm{HCG}}}$$

Consequently, the gain *K*_HDR_ can be described as

(7) $$K_{\mathrm{HDR}}=\frac{K_{D}K_{\mathrm{HCG}}B_{\mathrm{HCG}}}{B_{\mathrm{HDR}}}$$

The gain *K*_HDR_ of the fused HDR image depends on the adjusted HCG $K_{\mathrm{HCG}}^{\prime }$ and the adjusted digital gain $K_{D}^{\prime }$.

(8) $$K_{D}^{\prime }K_{\mathrm{HCG}}^{\prime }=K_{\mathrm{HDR}}=\frac{K_{D}K_{\mathrm{HCG}}B_{\mathrm{HCG}}}{B_{\mathrm{HDR}}}$$

As shown in Equation (8), the adaptive target gain is derived from the image brightness, the digital and HCG. Typically, *B*_HDR_ can be preset to half of the maximum value to balance detail preservation in both highlight and shadow regions [14].

For clarity, the specific calculation procedures for *K*_HCG_ and *K*_D_ are shown in Algorithm 1:
**Algorithm 1**: *K*_HCG_ and *K*_D_ Adjustment Pipeline**Input:** The brightness of the HCG mode: *B*_HCG_, The brightness of the HDR mode: *B*_HDR_, The analog gains of the HDR mode: *K*_HDR_, The max analog gains of the HCG mode: *K*_HCGMAX_**Output:** The digital gains: *K*_D_, The analog gains of the HCG mode: *K*_HCG_1 *K*_D_ = 1, *K*_HCG_ = 1;2 **if** *B*_HDR_ != *B*_HCG_ **then**3   *K*_D_ * *K*_HCG_ = (*B*_HCG_/*B*_HDR_) * *K*_HDR_;4   **if** (*K*_D_ * *K*_HCG_ <= *K*_HCGMAX_) **then**5     *K*_D_ = 1;6     *K*_HCG_ = (*B*_HCG_/*B*_HDR_) * *K*_HDR_;7   **end**8   **if** (*K*_D_ * *K*_HCG_ > *K*_HCGMAX_) **then**9     *K*_HCG_ = *K*_HCGMAX_;10     *K*_D_ = (*B*_HCG_/*B*_HDR_) * *K*_HDR_/(*K*_HCGMAX_);11   **end**12**end**13return *K*_D_, *K*_HCG_;

$K_{\mathrm{HCG}}^{\prime }$ and $K_{D}^{\prime }$ are obtained through Algorithm 1. Then the new analog gain $K_{\mathrm{HCG}}^{\prime }$ is transmitted to the sensor via Inter-Integrated Circuit (IIC) communication, while the digital gain $K\prime_{D}$ only needs to be updated for the subsequent calculation in Equation (3).

#### 2.2.2. Adaptive Threshold

This subsection focuses on the adaptive adjustment of the threshold *TH*, which directly controls the proportion of HCG and LCG components in the Clear HDR fusion process. As illustrated in Figure 1, the threshold *TH* ranges from 0 to 2*^N^* − 1, thereby influencing the readout noise level in the fused image. The fused image is entirely derived from the scaled LCG component when *TH* = 0, eliminating any transition from HCG to LCG and thus completely avoiding banding artifacts. However, in this case, the readout noise reaches its maximum value, equal to the LCG readout noise amplified by a factor of *K*_D_*K*_HCG_/*K*_LCG_. Conversely, banding artifacts are most pronounced when *TH* = 2*^N^* − 1, while the readout noise is minimized, corresponding only to the readout noise of the LCG portion involved in the fusion, scaled by *K*_D_*K*_HCG_/*K*_LCG_. As *TH* approaches 2*^N^* − 1, the buffer zone narrows, leading to more noticeable banding artifacts but further reducing readout noise.

Under the condition that the level of readout noise is acceptable, the minimum value of *TH* should be selected to minimize banding artifacts. Meanwhile, as indicated by Equation (4), this paper adopts the readout noise of the LCG component amplified by 2*^M^*^−*N*^ as the acceptable upper limit for readout noise.

The readout noise of the LCG mode, denoted as *L*_LCG_, is obtained through prior measurement. Based on the assumption of noise independence in the linear model [13], the readout noise of the Clear HDR fusion curve can be calculated by statistically analyzing the fused HDR data. Pixels with values above the threshold but below 2*^N^* − 1 are treated as a weighted average of the HCG and LCG components, with the number of such pixels denoted as *X*_M_. Pixels with values, greater than or equal to 2*^N^* − 1, are counted as *X*_H_. Utilizing the linear relationship between gain and readout noise, as well as the readout noise relationship before and after bit-depth extension, the readout noise of the Clear HDR fusion curve is given by

(9) $$L_{\mathrm{HDR}}=K_{D}\frac{\frac{X_{M}\cdot \frac{K_{\mathrm{HCG}}}{K_{\mathrm{LCG}}}\cdot L_{\mathrm{LCG}}}{2}+\frac{K_{\mathrm{HCG}}}{K_{\mathrm{LCG}}}\cdot X_{H}L_{\mathrm{LCG}}}{\left(X_{M}+X_{H}\right)}$$

When it is necessary to increase the image brightness by raising the gain, the term *K*_D_*K*_HCG_/*K*_LCG_ will correspondingly increase, leading to elevated readout noise. By reducing the proportion of LCG pixels in the image, the readout noise can be maintained at an acceptable level.

**Figure 2:**
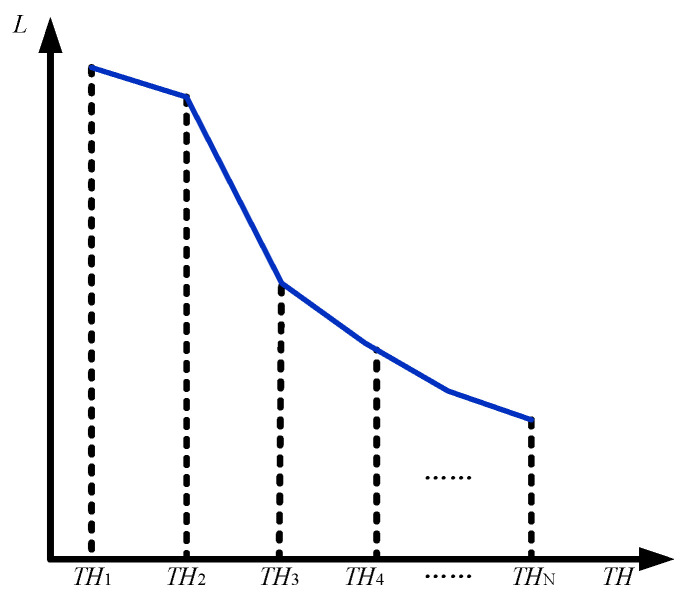
Relationship Between Readout Noise *L* and Threshold *TH*.

The proportion of the LCG component in the image can be adjusted by modifying the threshold *TH*. Since the distribution of pixel values in the image lacks a deterministic pattern, multiple thresholds *TH*_1_ to *TH_N_* are predefined, as illustrated in Figure 2. By evaluating the proportional contributions of each component in the Clear HDR fusion process at these thresholds, the readout noise levels *L*_HDR1_ to *L*_HDR*N*_ corresponding to each threshold can be calculated using Equation (9). During the adaptive adjustment of *TH*, the target threshold interval for *TH*’ is determined based on the desired readout noise level $L_{\mathrm{HDR}}^{\prime }$. Selecting the maximum value within this interval ensures that the actual readout noise $L_{\mathrm{HDR}}^{\prime }$ remains below the target noise level 2*^M^*^−*N*^*L*_LCG_. When the threshold reaches 2*^N^* − 1, the proportion of the HCG component in the fusion process is maximized, resulting in the lowest achievable readout noise within the adjustable range. If the noise level at this point still exceeds the desired level, it indicates that the noise in the LCG-based fused regions remains relatively high, whereas the noise in the HCG-based fused regions remains low. The pseudocode for adjusting *TH* is shown in Algorithm 2.
**Algorithm 2**: Threshold Adjustment Pipeline**Input:** The analog gains of the LCG mode: *K*_LCG_, The analog gains of the HCG mode: *K*_HCG_, The digital gains: *K*_D_, The Threshold array: *TH*[0 : N], the number of pixels with values between *TH* and 2*^N^* − 1: *X*_M_[0 : N], the number of pixels with values greater than or equal to 2*^N^* − 1: *X*_H_[0 : N], The readout noise of the LCG mode: *L*_LCG_, target readout noise *L*_T_**Output:** Threshold: *TH*1 *L*_HDR_ = 0, *i* = 0;2 **if** *i* < N **then**3   *L*_HDR_ = *K*_D_ * ((*X*_M_ * *K*_HCG_ * *L*_LCG_/(2 * *K*_LCG_)) + *K*_HCG_ * *X*_H_ * *L*_LCG_/*K*_LCG_)/(*X*_M_ + *X*_H_);4   **if** (*L*_HDR_ <= *L*_T_) **then**5     *TH* = *TH_i_*;6   **end**7   **if** (*L*_T_ <= *L*_HDR_) **then**8     *i* = *i* + 1;9   **else**10**end**11**if** (*i* = N) **then**12  *TH* = 2*^N^* − 1;13**end**14return *TH*;

Through iterative approximation of *TH* shown in Algorithm 2, the threshold satisfying the target readout noise level is ultimately obtained. This threshold, together with 2*^N^* − 1, defines the buffer zone. It should be noted that excessively increasing *TH* may introduce the aforementioned banding artifacts. Therefore, while constraining the readout noise level, the suppression effect of the buffer zone on banding artifacts must also be evaluated to determine a *TH* value that satisfies both requirements.

In summary, by adaptively adjusting the gain of the HCG component involved in the fusion and the threshold *TH*, it is possible to obtain fitting raw images with expected brightness and more rational fusion parameters under varying ambient lighting conditions. This enables the improved Clear HDR fusion model to ultimately produce high-quality HDR images.

## 3. Experimental Results

To validate the effectiveness and real-time capability of the proposed adaptive Clear HDR fusion algorithm, a series of experiments were conducted on an FPGA-based hardware platform. The Efinix T35F324I4 FPGA platform is adopted in this work as a case study, where fused HDR image data is transferred to a host computer via USB 3.0 interface for real-time visualization. The FPGA architecture employs Verilog HDL (Hardware Description Language) for the following computational modules. The Sony IMX664 CMOS image sensor serves as the front-end image acquisition module, featuring a Clear HDR mode that enables dual concurrent output of HCG and LCG image frames within a single readout cycle. The image bit depth is 12 bits in this case, i.e., *N* = 12. Based on the prevalence of the 16-bit image format, the case of *M* = 16 is adopted here, where the 12-bit data is upconverted to 16-bit depth.

To accommodate the limitation of standard displays, which are typically capable of rendering only 8-bit depth, all 16-bit images were processed in Adobe Photoshop with a standardized parameter set (detailed below) to ensure correct display. Adobe Photoshop 2022 is utilized as the software for post-processing. After opening the image in the software, go to the menu bar and select “Image > Adjustments > HDR Toning” [15]. Then, process the image with the following settings: Method: Local Adaptation; Edge Glow: Radius = 1 pixel, Strength = 0.1; Tone and Detail: Gamma = 1, Exposure = 0, Detail = 0%; Advanced: Shadow = 0%, Highlight = 0%, Vibrance = 0%, Saturation = 0%; Toning Curve and Histogram: Default (no adjustment). The uniformity of these parameters ensured that no additional variables were introduced. In contrast, such processing was omitted for the 12-bit LCG and HCG source images, as the fusion quality assessment remains unaffected by their display accuracy on 8-bit monitors.

The IMX664 sensor’s registers are first configured via the Inter-Integrated Circuit (I2C) protocol to activate its Clear HDR mode, enabling simultaneous output of HCG and LCG image streams. The fusion curve is optimized by modulating the core parameters governing Clear HDR synthesis to achieve perceptually refined HDR output. The processed image data is subsequently transferred via USB 3.0 hardware interface to a host computer for real-time visualization and analysis.

**Figure 3:**
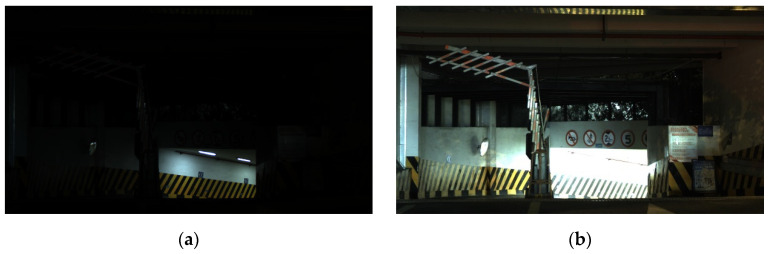
Source images of the garage entrance night scene for HDR fusion (**a**) LCG image; (**b**) HCG image.

The proposed adaptive algorithm was evaluated and compared against the HDR fusion algorithms by Liu [8], Li [9], and Mertens [7], along with the method by Xu [12] that also employs the Clear HDR algorithm. Figure 3a and Figure 3b, respectively, show the original LCG and HCG images of the garage entrance night scene, which were simultaneously output by the sensor and used for HDR fusion. As can be observed, in the LCG image, while the details of the illuminated wall are well-preserved, the remaining areas are too dark, resulting in a loss of detail. In contrast, the HCG image has an overall higher brightness and retains most of the information in the darker regions. However, the illuminated wall area is overexposed, leading to washed-out details. Individually, neither of these two images can satisfactorily represent the actual scene.

**Figure 4:**
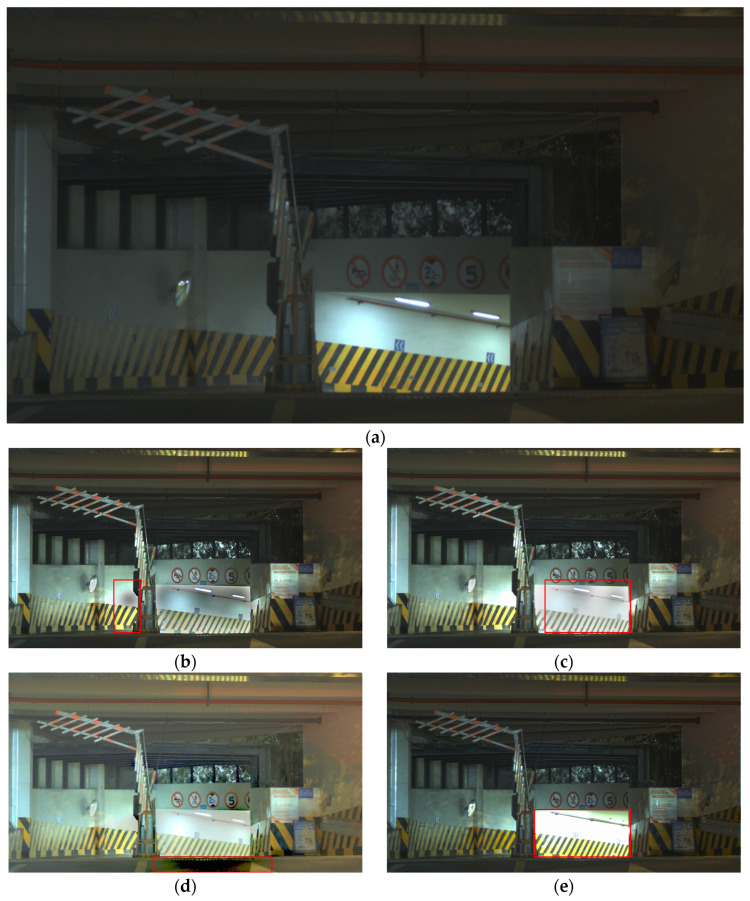
HDR fusion results for the garage entrance night scene using different algorithms: (**a**) Proposed, (**b**) Li, (**c**) Liu, (**d**) Mertens, and (**e**) Xu. (All native 16-bit results are uniformly converted to 8-bit for display).

For the scene shown in Figure 3, Figure 4a presents the garage entrance night scene generated by our proposed HDR fusion algorithm, while (b)–(e) Figure 4b–e display the results obtained by the algorithms of Li, Liu, Mertens, and Xu, respectively. All algorithms were fed with the same 12-bit LCG and HCG source images shown in Figure 3, producing 16-bit outputs that were uniformly converted to 8-bit for correct display on standard monitors. The proposed HDR fusion result appears more visually natural, as the fused data preserves the original sensor response curve, thereby retaining greater flexibility during HDR tone mapping. In contrast, the algorithms by Li and Liu exhibit unnatural shadow regions overall, with noticeable halo artifacts particularly along edges between bright and dark areas, as highlighted in the red boxes in Figure 4b,c. The Mertens’ algorithm shows significant improvement over Li and Liu, with relatively smooth brightness transitions. However, severe banding artifacts appear at the bottom of the image as highlighted in the red box in Figure 4d. The fusion algorithm by Xu relies on a fixed ratio between high and low gain levels, limiting its adaptability to scenarios with varying gain relationships. As a result, it fails to adequately suppress overexposure as highlighted in the red box in Figure 4e.

**Figure 5:**
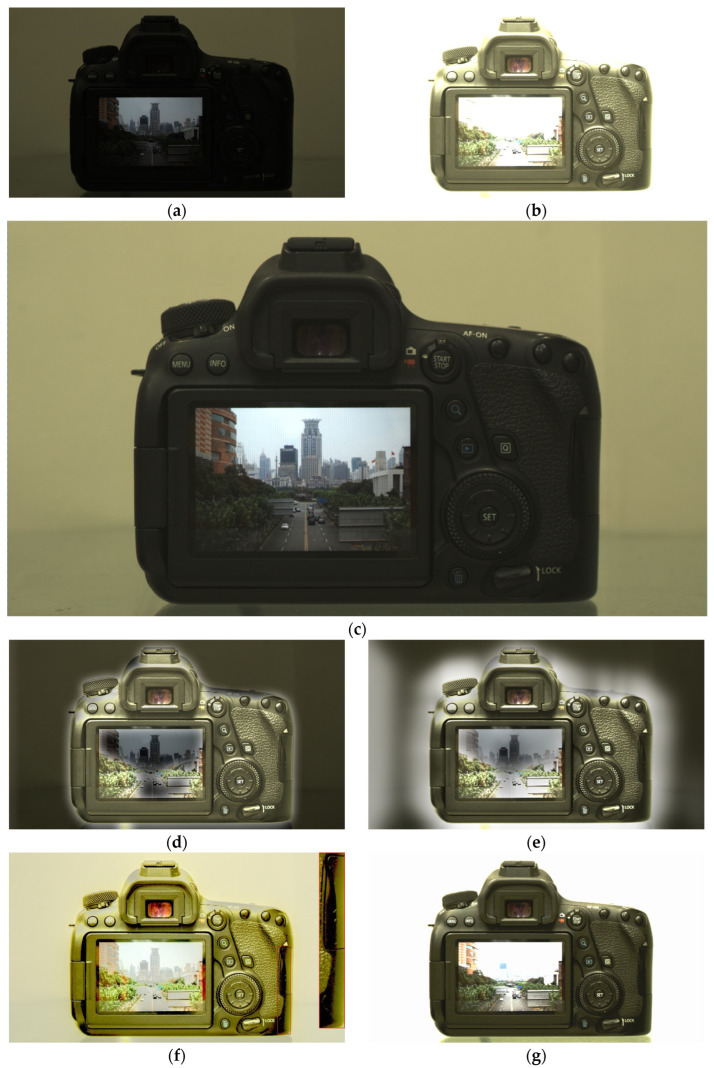
Source images of the camera with a lit screen for HDR fusion (**a**) LCG image, (**b**) HCG image and the corresponding HDR fusion results using different algorithms: (**c**) Proposed, (**d**) Li, (**e**) Liu, (**f**) Mertens, and (**g**) Xu. (All native 16-bit HDR fusion results are uniformly converted to 8-bit for display).

**Figure 6:**
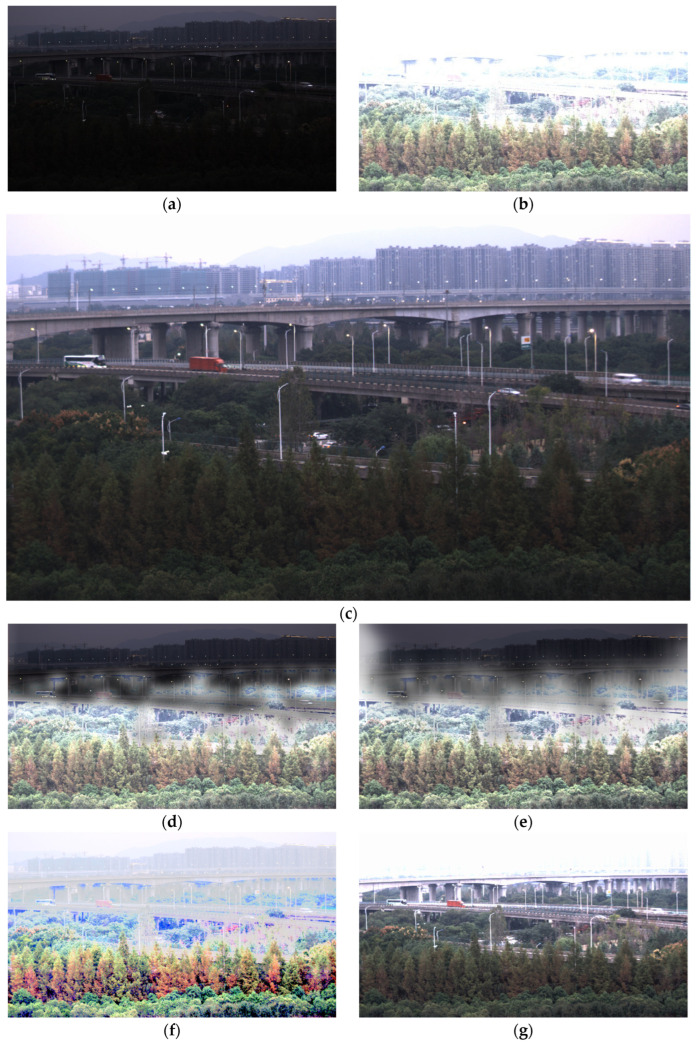
Source images of the interchange landscape for HDR fusion (**a**) LCG image, (**b**) HCG image and the corresponding HDR fusion results using different algorithms: (**c**) Proposed, (**d**) Li, (**e**) Liu, (**f**) Mertens, and (**g**) Xu. (All native 16-bit HDR fusion results are uniformly converted to 8-bit for display).

To evaluate the robustness of these HDR fusion algorithms, we introduced additional test scenarios. Figure 5 and Figure 6 present the HDR fusion results generated by our algorithm and the comparative algorithms for two distinct scenarios: a camera with a lit screen and an interchange landscape, respectively.

As shown in Figure 5, Liu’s algorithm exhibits unnatural shadow regions overall, with noticeable halo artifacts along both the upper edge of the image and the top contour of the camera. Li’s algorithm shows some improvement in reducing halo effects around the camera, but the brightness balance between the ambient lighting and the camera appears inconsistent, resulting in an unnatural representation of the main subject. Additionally, obvious halos are present around the content displayed on the camera screen. Mertens’ algorithm produces more natural results compared to the former two; however, color distortion is observable, and banding artifacts occur in red-boxed area. Li, Liu and Mertens’ algorithms demonstrate unnatural transitions at the junction of the screen borders. The issue with Xu’s method persists, as its reliance on fixed fusion parameters leads to inadequate suppression of overexposed regions and loss of background context. In contrast, the HDR image generated by our algorithm successfully preserves the camera’s external features, on-screen content, and background information without introducing any halo artifacts or color cast. This demonstrates that our algorithm achieves satisfactory HDR fusion results for the camera scene.

As shown in Figure 6, halo artifacts along brightness transition regions remain a major issue for both Liu’s and Li’s algorithms. Although Mertens’ algorithm avoids halo artifacts, the white balance of the fused image is noticeably distorted—a phenomenon not observed in other traditional HDR fusion algorithms. Xu’s algorithm successfully suppresses overexposure in the tree areas, but due to its fixed fusion parameters, overexposure in the building regions is not adequately controlled. In contrast, the HDR image produced by our algorithm successfully preserves the darker vegetation in the foreground, as well as distant high-rises and mountains, while introducing no halo artifacts, color cast, and maintaining smooth brightness transitions. These results demonstrate that our algorithm also achieves satisfactory HDR fusion performance for the interchange landscape scene.

Based on Figure 4, Figure 5 and Figure 6, the algorithms proposed by Li, Liu, and Mertens demonstrate satisfactory performance in the garage entrance night scene, but their fusion results become increasingly unnatural in the camera and interchange landscape scenes. The primary reason is that these algorithms impose stringent requirements on the source images: details in the scene must be well preserved in the input exposures to achieve a natural fused result. When the LCG image is too dark to adequately represent highlight details, or when the HCG image is too bright to capture shadow details effectively, these conventional HDR fusion methods produce visibly artificial outcomes. For instance, in the Camera scene, the background brightness captured in the LCG image is significantly darker than the camera body brightness recorded in the HCG image. This discrepancy prevents conventional algorithms from achieving smooth transitions during fusion. Similarly, in the Street scene, the dark trees captured in the HCG image are considerably brighter than the buildings recorded in the LCG image, leading to unnatural transitions in the fusion results produced by conventional methods. In contrast, the proposed method leverages the intrinsic relationship between the LCG and HCG sources, enabling effective utilization of both underexposed and overexposed image data to generate a more natural fused image.

Subjective observation can only provide a general assessment of the merits of a fusion algorithm based on personal preference, while analyzing the objective parameters of images before and after fusion across three scenarios can offer a concrete basis for comparing and selecting algorithms. Among these, the metricMI [16] parameter reflects the amount of information in the fused image derived from the input image sequence, indicating, to some extent, the extent to which the fusion algorithm utilizes and retains the original data. As shown in Table 1, a higher metricMI indicates that the fused image contains more information derived from the input image sequence. The values in bold represent the best results for each scenario. By comparing the five algorithms horizontally, it can be observed that the proposed method achieves nearly the best metricMI across all three scenarios. This is attributed to its ability to more effectively utilize overexposed or underexposed images in the fusion process.

**Figure 7:**
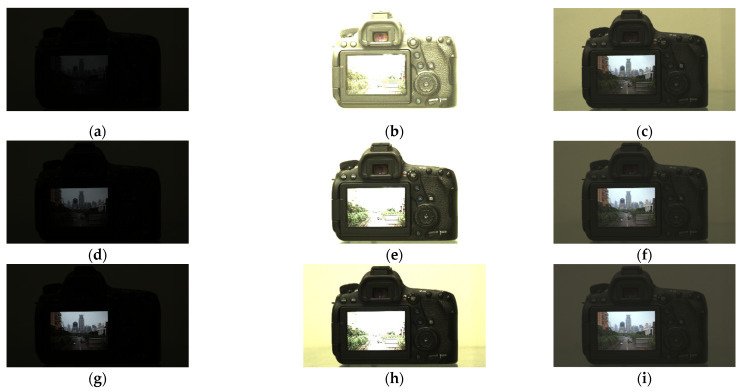
Varying screen brightness levels (15, 45, and 100 Lux from top to bottom) result in corresponding changes in the LCG (**a**,**d**,**g**), HCG (**b**,**e**,**h**) and HDR (**c**,**f**,**i**) images.

Based on the fundamental fusion model, the adaptive fusion model proposed in this paper can dynamically adjust fusion parameters in real-time according to scene brightness, thereby ensuring the capture of complete image information for fusion. Figure 7 illustrates a scenario where the brightness of the subject changes, simulated by adjusting the screen brightness of the camera. The varying brightness levels of the LCG fusion sources represent different subject brightness levels, with screen luminance of 15 Lux, 45 Lux, and 100 Lux from top to bottom. Faced with changing subject brightness, the algorithm adaptively increases *K*_HCG_ to produce fused images with appropriate brightness—specifically, *K*_HCG_ values of 118.75, 36.875, and 18 from top to bottom. As shown in Figure 7, despite noticeable variations in the LCG (a, d, g) and HCG (b, e, h) caused by different screen brightness levels, our adaptive algorithm achieves favorable HDR fusion results in all cases, as evidenced in Figure 7c,f,i. Both the pre-fusion source images and the resulting HDR images demonstrate that, without altering the exposure time, adaptive adjustment of *K*_HCG_ enables the acquisition of suitable source data for generating high-quality fused images.

**Figure 8:**
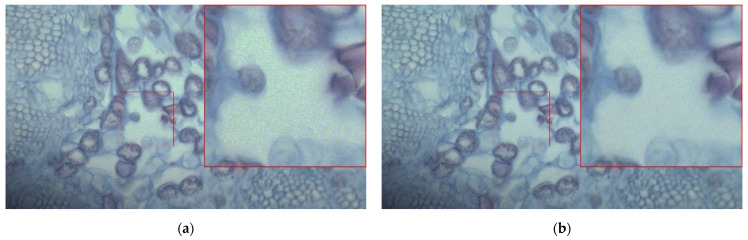
HDR fusion microscopic images of a corn slice (**a**) without and (**b**) with the buffer module.

As illustrated in Equation (3), a buffer is introduced between the threshold *TH* and 2*^N^* − 1 to mitigate the HDR image banding artifacts caused by amplified noise discontinuities during the transition from HCG to the amplified LCG. A comparative experiment is conducted evaluating the algorithm with and without this buffer component to validate the functionality of the buffer layer. Figure 8a and Figure 8b display the HDR fusion images of a corn slice without and with the buffer module, respectively. By magnifying the identical region within the central red box, it is clearly observed that the result from the algorithm without the buffer exhibits noticeable banding artifacts, whereas the result incorporating the buffer appears significantly smoother. This demonstrates that the introduction of the buffer effectively mitigates the banding artifacts in the HDR fused image caused by noise discontinuities.

**Figure 9:**
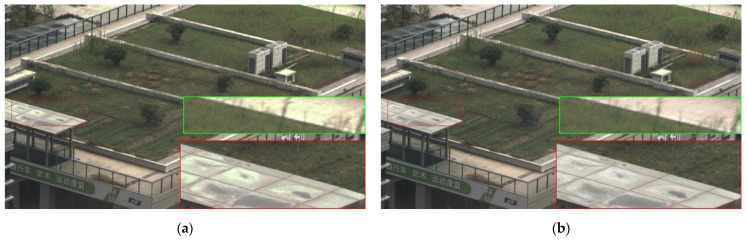
HDR fusion images of a roof (outdoor scene) (**a**) without and (**b**) with the buffer module.

**Figure 10:**
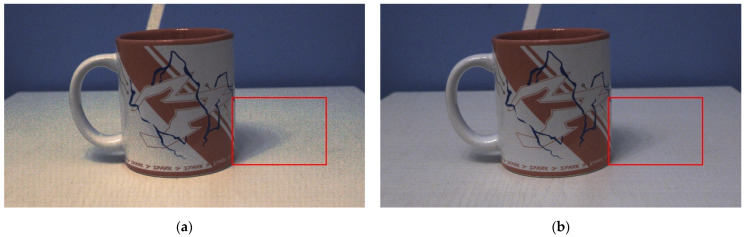
HDR fusion images of a mug (indoor scene) (**a**) without and (**b**) with the buffer module.

Unlike the relatively uniform lighting conditions in microscopy, everyday imaging scenarios often involve complex lighting variations, making banding artifacts more likely to occur and more visually noticeable. Figure 9 and Figure 10 present the HDR fusion images of a roof (outdoor scene) and a mug (indoor scene) without and with the buffer module, respectively. As shown in Figure 9, it can be observed that the banding artifacts in the canopy area (marked by the red box on the left) and the road surface (marked by the green box on the upper right) are significantly improved. The highlighted area within the red box in Figure 10a exhibits noticeable banding and color shift at the transition between high-gain and low-gain regions. After applying the buffer zone, these artifacts are significantly reduced, and the desktop color remains consistent across the transition, as shown in Figure 10b. These examples collectively demonstrate that introducing a buffer effectively mitigates the image banding artifact, thereby creating the condition and possibility for subsequent image processing.

Finally, A runtime comparison was also performed to ensure a comprehensive evaluation of all algorithms. For algorithms implemented and deployed on an FPGA, the execution follows a pipelined architecture. Therefore, the runtime depends solely on the image size and the operating clock frequency of the FPGA chip. Let *H* and *W* denote the number of pixels along the height and width of the image, respectively, *c* denote the number of channels, and *f* denote the clock frequency. Then, the execution time *t* of the fusion algorithm on the FPGA can be expressed as [12]

(10) $$t=\frac{H\times W}{c\times f}$$

To ensure fairness in the comparative experiments, both Xu’s method and the proposed method in this paper adopted identical parameters, i.e., an image size of 2688 × 1520 pixels, the sensor operating in 4-channel mode, and an FPGA operating frequency of 100 MHz. By substituting these data into Equation (10), the execution time for both Xu’s method and the proposed method is calculated as 0.0102144 s per frame, which demonstrates 97.83% faster processing compared to conventional methods (Mertens, Liu, and Li) as benchmarked in Table 2. In other words, our proposed HDR fusion algorithm is capable of processing 2688 × 1520 resolution video streams at 46 frames per second, thus enabling real-time video processing. This breakthrough performance is attributed to both FPGA-optimized parallel computing architecture and a streamlined Clear HDR fusion pipeline requiring.

## 4. Discussion

First, the proposed algorithm is implemented on an FPGA, which ensures that the processing time for each set of images to be fused remains constant and extremely short. For the resolution of 2688 × 1520 images used in this work, the execution time is 0.0102144 s, allowing the hardware platform to process up to 97 sets of images per second without causing data congestion. If further improvement in processing speed is desired in the future, it can be achieved simply by increasing the FPGA operating frequency or expanding the number of sensor channels, without being limited by the algorithm itself. This fundamentally enables the proposed algorithm to outperform the methods of Li [9], Liu [8], and Mertens [7] in terms of real-time performance.

Second, the algorithm fully leverages the 16-bit data format, extending 12-bit source images to a fused 16-bit output while preserving a linear response curve. Maintaining this linear response curve produces more natural fusion results compared to the algorithms of Li [9], Liu [8], and Mertens [7]. Moreover, the core idea of expanding the bit depth to 16 bits results in a genuine increase in dynamic range for the fused images. Compared to Xu’s algorithm [12], although the expanded bit depth may be less convenient for direct viewing, this drawback is negligible given that both algorithms output images beyond 8 bits. In specialized fields such as astrophotography, which require extensive post-processing, preserving a higher dynamic range is actually advantageous for subsequent adjustments.

Finally, based on the fusion model proposed in this paper, a mechanism for adjusting fusion parameters has been investigated, enabling the fusion algorithm to adaptively modify its parameters under varying illumination conditions for proper operation. This aspect was not deeply explored in Xu’s algorithm [12], which explains why his algorithm sometimes produces overly bright or dark fusion results in certain scenarios.

In summary, this paper ultimately presents an HDR fusion algorithm with adaptive parameter adjustment, low latency, and high linearity. The fused images obtained through this algorithm genuinely expand the image bit depth, effectively achieving the original design goal of real-time HDR video stream output.

## 5. Conclusions

Based on the Clear HDR functionality, this study not only achieves high dynamic range (HDR) in fused images but also simplifies the capture process, enabling real-time HDR video streaming. By adaptively adjusting fusion parameters according to target brightness and readout noise levels, the method is particularly suitable for dynamically changing video streams. The incorporation of a buffer zone in the proposed fusion model significantly mitigates potential banding artifacts. The algorithm is implemented on an FPGA platform, leveraging its high-speed parallel processing capability to achieve at least a 97% improvement in processing speed compared to conventional HDR fusion methods, thereby realizing adaptive real-time video fusion. The fused images preserve a higher dynamic range authentically, enhancing flexibility in post-processing and providing a viable alternative for acquiring HDR images in professional photography applications.

## Figures and Tables

**Table 1:** metricMI of HDR algorithms for different scenes.

Image	Liu	Li	Mertens	Xu	Proposed
Garage entrance	1.2863	1.1608	0.9285	1.3443	**1.3948**
Camera	0.6911	0.4097	0.6970	1.2879	**1.3647**
Interchange landscape	0.8117	0.6002	0.7567	1.2837	**1.3454**

Note. The values in bold represent the best results for each scenario.

**Table 2:** Execution time of HDR algorithms for different scenes (Image size: 2688 × 1520; Unit: seconds).

Image	Liu	Li	Mertens	Xu	Proposed
Garage entrance	8.683543	4.146555	0.471591	**0.0102144**	**0.0102144**
Camera	8.347134	5.432010	0.439001	**0.0102144**	**0.0102144**
Interchange landscape	9.167345	4.231708	0.519887	**0.0102144**	**0.0102144**
Average	8.732674	4.603424	0.476826	**0.0102144**	**0.0102144**

Note. The values in bold represent the best results for each scenario.

## Data Availability

Data are contained within the article.

## Abbreviations

The following abbreviations are used in this manuscript:

HDR	High Dynamic Range
FPGA	Field Programmable Gate Array
HCG	High Conversion Gain
LCG	Low Conversion Gain
SIFT	Scale-Invariant Feature Transform
